# A Novel Ocellatin-P1 Isoform from *Leptodactylus labyrinthicus* Frog Skin Secretion: Purification, Biological Properties and Three-Dimensional Structure

**DOI:** 10.3390/ijms27083658

**Published:** 2026-04-20

**Authors:** César Augusto Prías-Márquez, Eliane Santana Fernandes Alves, Carlos José Correia de Santana, Osmindo Rodrigues Pires Júnior, Eduardo Maffud Cilli, Fabiano José Queiroz Costa, Alice da Cunha Morales Álvares, Sonia Maria de Freitas, Isabel de Fátima Correia Batista, Rafael Marques Porto, Isabelle S. Luz, Ricardo B. Azevedo, João Paulo Stawiarski Miranda, Henrique de Oliveira Noronha, Marco Antônio Damasceno Faustino, Felipe da Silva Mendonca de Melo, Alexandra Maria dos Santos Carvalho, Izabela Marques Dourado Bastos, Wagner Fontes, Aline L. Oliveira, Luciano M. Lião, Mariana S. Castro

**Affiliations:** 1 Laboratório de Toxinologia, Departamento de Ciências Fisiológicas, Instituto de Ciências Biológicas, Universidade de Brasília, Brasília 70910-900, DF, Brazil; caprias@gmail.com (C.A.P.-M.); carlosjcsantana@gmail.com (C.J.C.d.S.); osmindo@unb.br (O.R.P.J.); 2 Laboratório de Ressonância Magnética Nuclear, Instituto de Química, Universidade Federal de Goiás, Goiânia 74001-970, GO, Brazil; eliane_ufg@yahoo.com.br (E.S.F.A.); lucianoliao@ufg.br (L.M.L.); 3 Departamento de Bioquímica e Tecnologia Química, Instituto de Química, Universidade Estadual de São Paulo, Araraquara 14800-900, SP, Brazil; eduardo.cilli@unesp.br; 4 Laboratório Central de Saúde Pública (LACEN), Brasília 70830-010, DF, Brazil; fj3unb@gmail.com; 5 Laboratório de Biofísica, Departamento de Biologia Celular, Universidade de Brasília, Brasília 70910-900, DF, Brazil; pharmalice@gmail.com (A.d.C.M.Á.); nina@unb.br (S.M.d.F.); 6 Unidade de Sequenciamento de Peptídeos e Proteínas, Instituto Butantan, São Paulo 05503-900, SP, Brazil; isabel.batista@butantan.gov.br (I.d.F.C.B.); rafael.porto@butantan.gov.br (R.M.P.); 7 Laboratório de Bioquímica e Química de Proteínas, Departamento de Biologia Celular, Instituto de Ciências Biológicas, Universidade de Brasília, Brasília 70910-900, DF, Brazil; isabelle.sluz@gmail.com (I.S.L.); wagnerf@unb.br (W.F.); 8 Departamento de Genética e Morfologia, Instituto de Ciências Biológicas, Universidade de Brasília, Brasília 70910-900, DF, Brazil; razevedo@unb.br; 9 Insituto de Física, Universidade de Brasília, Brasília 70910-900, DF, Brazil; jpstawiarski.hs@gmail.com (J.P.S.M.); henrique.noronha@yahoo.com.br (H.d.O.N.); pocotoin@gmail.com (M.A.D.F.); 10 Laboratório da Interação Patógeno-Hospedeiro, Departamento de Biologia Celular, Instituto de Ciências Biológicas, Universidade de Brasília, Brasília 70910-900, DF, Brazil; felipe.sdmm@gmail.com (F.d.S.M.d.M.); alexandrasantos22@hotmail.com (A.M.d.S.C.); dourado@unb.br (I.M.D.B.); 11 Laboratório de Ressonância Magnética Nuclear, Instituto de Química, Universidade de Brasília, Brasília 70910-900, DF, Brazil; aloliveira@unb.br

**Keywords:** anurans, skin secretion, *Leptodactylus labyrinthicus*, antimicrobial peptides, ocellatins, therapeutic properties, NMR structure

## Abstract

A novel ocellatin-P1 isoform was isolated and purified from the skin secretion of the pepper frog *Leptodactylus labyrinthicus*. The crude skin secretion was fractionated by reversed-phase high-performance liquid chromatography (RP-HPLC) using a C_8_ column and the peptide was subsequently purified on a reversed-phase C_18_ column. Ocellatin-LB3 (as this isoform was named) was chemically sequenced by Edman degradation. This peptide is a linear C-terminally amidated molecule composed of 25 amino acid residues: ^1^GLLDTLKGAAKNVVGGLASKVMEKL^25^-NH_2_. Synthetic ocellatin-LB3 was active against *Escherichia coli*, *Klebsiella pneumoniae* and *Pseudomonas aeruginosa* and inactive against *Staphylococcus aureus*, *Staphylococcus epidermidis* and *Enterococcus faecalis*. In addition, the peptide reduced the *Trypanosoma cruzi* infection in L6 cells. At 64 µM it did not reduce erythrocytes or polymorphonuclear leukocytes, but did reduce mononuclear leukocyte counts, as detected by flow cytometry. No hemolytic activity was observed in red blood cells even at 128 µM. The peptide exhibited limited antiproliferative activity against MCF-7 and HeLa tumor cells at 128 µM. Pre-incubation with the peptide appeared to enhance N-formylmethionine-leucyl-phenylalanine (fMLP)-induced migration, indicating a potential additive or synergistic effect on human neutrophils. The three-dimensional structure of ocellatin-LB3 was investigated by circular dichroism (CD) and nuclear magnetic resonance (NMR). In the presence of sodium dodecyl sulfate (SDS), the peptide adopts an α-helical structure spanning residues Leu^3^–Lys^24^, which remains largely preserved even at 95 °C. NMR Hydrogen/Deuterium (H/D) exchange experiments suggest that ocellatin-LB3 adopts a preferential orientation when interacting with SDS micelles. Based on the similarity among ocellatins, and on the physicochemical and structural properties of this peptide, a possible membrane-mediated mode of action is proposed, although this remains to be experimentally validated.

## 1. Introduction

The skin of amphibians is a rich source of pharmacologically active molecules with neurotoxic, myotoxic, vasoconstrictor, hallucinogenic, hypotensive, antinociceptive, hormone-releasing, antimicrobial, and cytotoxic activities [1,2,3,4,5,6]. Among these compounds, antimicrobial peptides (AMPs) are released as part of a first-line defense mechanism against environmental pathogens [7,8]. Anuran skin has attracted considerable interest as a source of new bioactive molecules because of the remarkable peptide diversity found in these animals, as well as its potential for the development of new antibiotics to help address the current antibiotic resistance crisis [9,10].

Ocellatins are a group of peptides isolated from the skin secretions of frogs belonging to the Leptodactylidae family. They are highly conserved linear peptides comprising between 21 and 32 amino acid residues. Most of these peptides have amidated C-termini and display antimicrobial activity against a variety of bacteria and fungi, and several also show hemolytic activity [10,11,12,13,14,15,16,17,18,19,20,21]. A nomenclature for this class of peptides was proposed by Conlon [22] and is adopted in the present work. Ocellatin-P1 (originally named pentadactylin) is a poorly hemolytic peptide isolated from the skin secretions of *Leptodactylus pentadactylus* and *Leptodactylus labyrinthicus*. It is active against several Gram-negative (*Escherichia coli*, *Klebsiella pneumoniae*, and *Pseudomonas aeruginosa*) and Gram-positive (*Staphylococcus aureus*, *Staphylococcus epidermidis*, *Enterococcus faecalis*, and *Streptococcus* group B) bacterial strains. It contains 25 amino acid residues, has a positive net charge at neutral pH, and is predicted to adopt an α-helical structure upon contact with the cell membrane [13,23]. In particular, understanding how sequence variations affect structure and membrane interaction is essential for elucidating structure–function relationships within the ocellatin family. Therefore, this study aims not only to characterize a novel isoform, but also to investigate its structural behavior in membrane-mimetic environments.

*L. labyrinthicus* (Spix, 1824), commonly known as the pepper frog, is a large-sized species that inhabits savannah enclaves and open habitats in dry and moist tropical forests. It can be found in the Cerrado and Caatinga regions of Brazil, as well as in eastern Paraguay, Bolivia, and northern Argentina, at elevations of up to 1000 m. It is a highly adaptable species and an effective colonizer of man-made habitats and formerly closed environments [24,25].

This study describes a new member of the ocellatin family obtained from the skin secretion of *L. labyrinthicus*. It also includes the characterization of its biological properties and its structural analysis by CD and NMR.

## 2. Results

**Figure 1:**
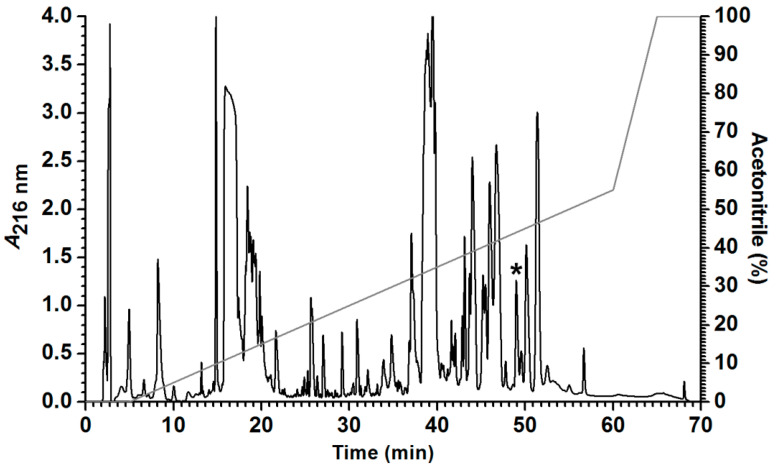
Chromatographic profile of *L. labyrinthicus* crude skin secretion fractionated by RP-HPLC on a C_8_ column (Shim-pack CLC-C8(M) 150 x 4.6 mm, Shimadzu Corporation, Kyoto, Japan). Fractions were manually collected while monitoring absorbance at 216 nm, using a linear gradient of 0.1% TFA/acetonitrile. The fraction eluted at 49.5 min (*) corresponds to ocellatin-LB3 and was subsequently purified on a C_18_ column (Shim-pack VP-ODS 150 x 4.6 mm, Shimadzu Corporation, Kyoto, Japan).

**Figure 2:**
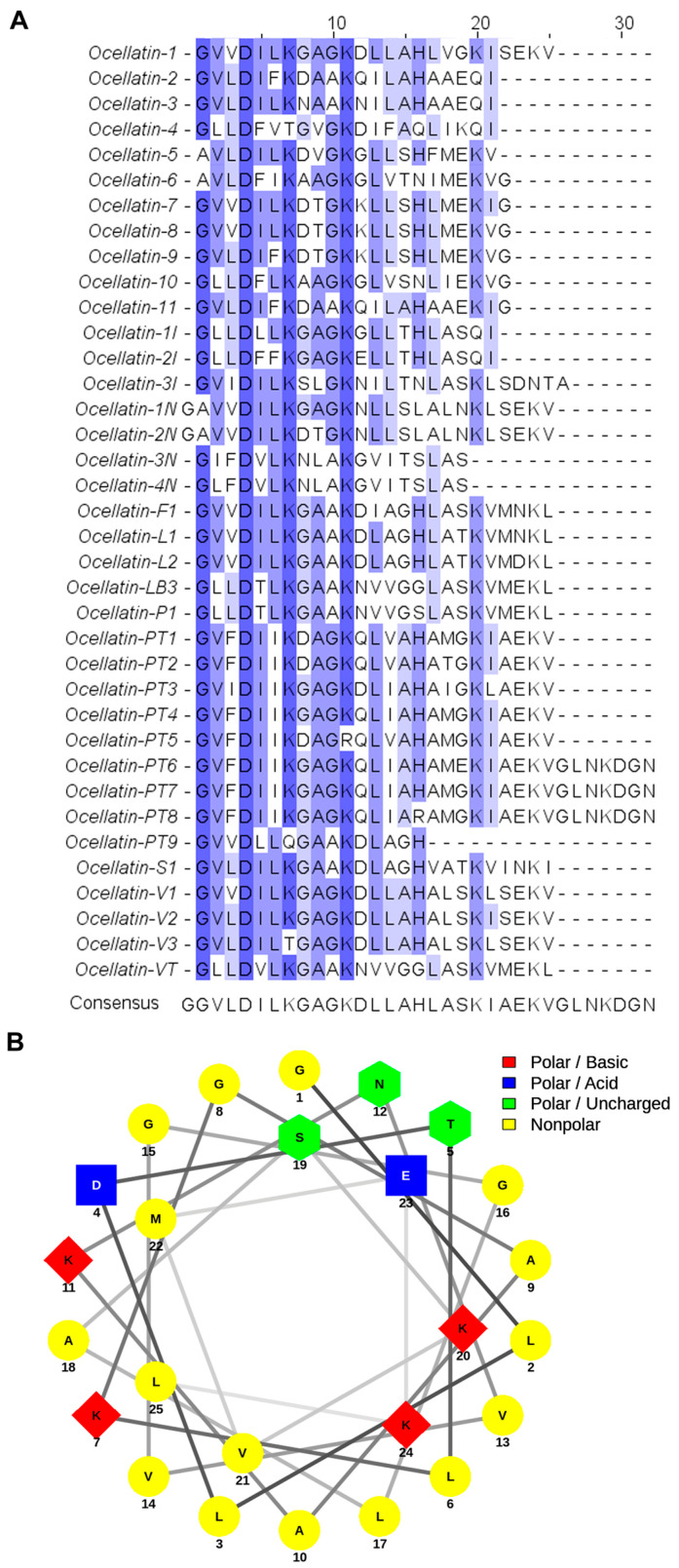
(**A**) Multiple sequence alignment of selected ocellatins generated using Clustal Omega. A consensus sequence is presented at the bottom of the figure. Residues that agree with the consensus are shaded, and halftones indicate the degree of agreement at each position, with darker shading representing greater agreement. Ocellatin-LB1 and ocellatin-LB2 were not included in the alignment because they actually represent fragments of ocellatin-F1. (**B**) Schiffer–Edmundson projection showing the distribution of amino acid residues in the α-helix of ocellatin-LB3.

*Leptodactylus labyrinthicus* produced a rich and complex skin secretion, yielding approximately 47 peaks after C_8_ RP-HPLC fractionation. One fraction, eluting at approximately 49.5 min (45% solvent B, Figure 1), displayed antibacterial activity. This fraction was then pooled, and its peptide components were purified by C_18_ RP-HPLC, yielding a peptide with a monoisotopic mass of 2510.47 Da. Edman degradation revealed the following linear sequence: ^1^GLLDTLKGAAKNVVGGLASKVMEKL^25^, which is identical to that of the previously isolated leptodactylid peptide ocellatin-P1 (pentadactylin) [13], except for the substitution of glycine for serine at position 16. In accordance with the nomenclature proposed by Conlon [22], and considering that two additional peptides have been reported for this species [20], this peptide was named ocellatin-LB3. Figure 2A presents the multiple alignment of ocellatin-LB3 with peptides of the ocellatin family. A Schiffer–Edmundson helical wheel projection of this peptide is shown in Figure 2B.

Sequence-based calculations and the experimentally determined molecular mass differed by approximately 1 Da (2511.45 vs. 2510.47 Da monoisotopic mass, respectively), suggesting the presence of a C-terminal amidation in the molecule. This was corroborated by analysis of methylated acidic groups through Fischer-Speier esterification.

Ocellatin-LB3 showed inhibitory activity against the Gram-negative bacteria *E. coli*, *K. pneumoniae*, and *P. aeruginosa* at low concentrations (MICs ranging from 4 to 64 µM) and against *S. epidermidis* only at the highest concentration tested (MIC = 128 µM). Nevertheless, it was not active against *S. aureus* or *E. faecalis* at any tested concentration (Table 1). For comparison, magainin 2 was used as a model AMP and displayed MIC values of 32 µM against *P. aeruginosa* and *K. pneumoniae* under the same experimental conditions.

**Figure 3:**
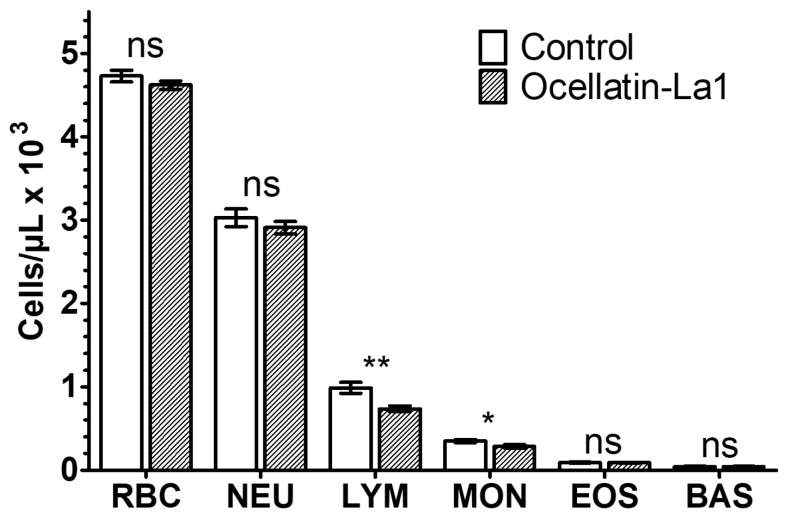
Effect of synthetic ocellatin-LB3 on red and white blood cell counts after 30 min of incubation with saline (control) or 64 µM ocellatin-LB3. Data are presented as the mean ± standard deviation of triplicate measurements for each treatment. RBC, red blood cells; NEU, neutrophils; LYM, lymphocytes; MON, monocytes; EOS, eosinophils; BAS, basophils. ns, not significant; * *p* < 0.05; ** *p* < 0.01.

Ocellatin-LB3 did not cause a significant reduction in polymorphonuclear leukocytes in blood, including neutrophils (NEU), eosinophils (EOS), and basophils (BAS), after 30 min of incubation at 64 µM (unpaired *t*-test, *p* > 0.05 for NEU, EOS, and BAS). However, significant reductions were observed in mononuclear leukocytes, namely monocytes (MON) and lymphocytes (LYM) (unpaired *t*-test, *p* < 0.05 for MON and *p* < 0.01 for LYM). These effects remained unchanged after 90 min of incubation. For LYM, but not MON, the effect was already observed immediately after incubation (0 min) (unpaired *t*-test, *p* < 0.01). Finally, no significant reduction in red blood cell (RBC) counts was observed after 0, 30, or 90 min of incubation with 64 µM ocellatin-LB3. Consistently, the hemolysis assay showed no lytic activity, even at twice this concentration (128 µM, the highest concentration tested). Figure 3 summarizes the results obtained by cytometric cell counting after 30 min of incubation.

**Figure 4:**
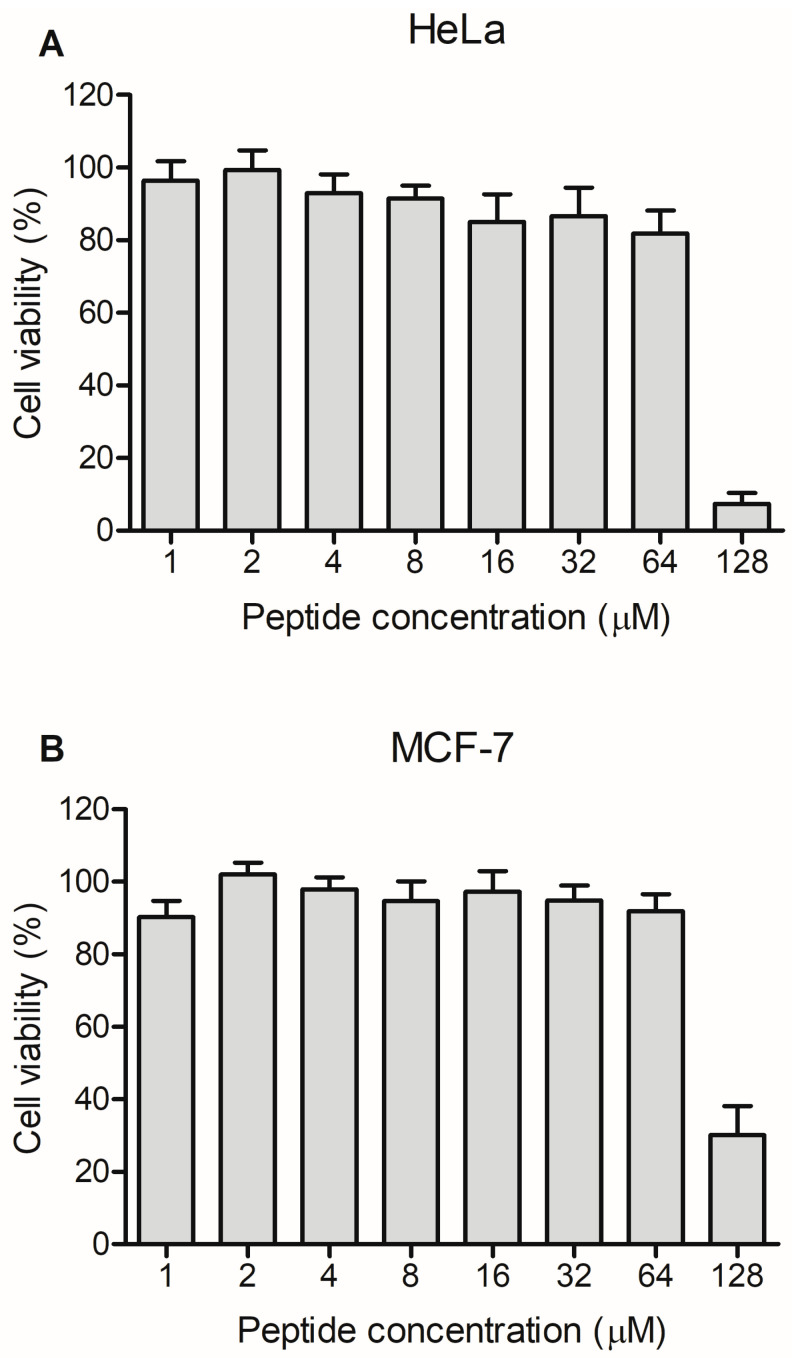
Antiproliferative activity of synthetic ocellatin-LB3 against the tumor cell lines (**A**) HeLa (human cervical adenocarcinoma) and (**B**) MCF-7 (human breast adenocarcinoma).

Ocellatin-LB3 showed no antiproliferative activity against HeLa and MCF-7 tumor cell lines at the tested concentrations, except at the highest concentration (128 µM), which resulted in approximately 93% inhibition of HeLa cell proliferation and approximately 70% inhibition of MCF-7 cell proliferation (Figure 4).

**Figure 5:**
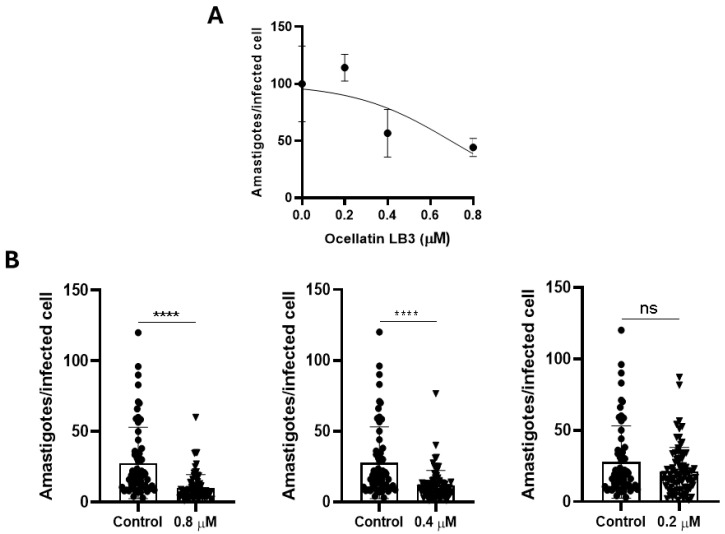
Anti-*T. cruzi* activity of synthetic ocellatin-LB3. (**A**) Dose–response curve showing the percentage inhibition of infection at different peptide concentrations. (**B**) Number of amastigotes per infected cell after treatment with the peptide at different concentrations (0.8, 0.4, and 0.2 µM). Unpaired *t*-test; **** *p* < 0.0001; ns, not significant.

The in vitro activity of ocellatin-LB3 was evaluated in L6 cells infected with *Trypanosoma cruzi*. The cells were treated 24 h after infection, when the internalized parasites had differentiated into the replicative amastigote form. Ocellatin-LB3 reduced the infection rate in a dose-dependent manner (Figure 5A). Significant reductions in the number of amastigotes per infected cell were observed at concentrations of 0.4 and 0.8 µM (Figure 5B).

**Figure 6:**
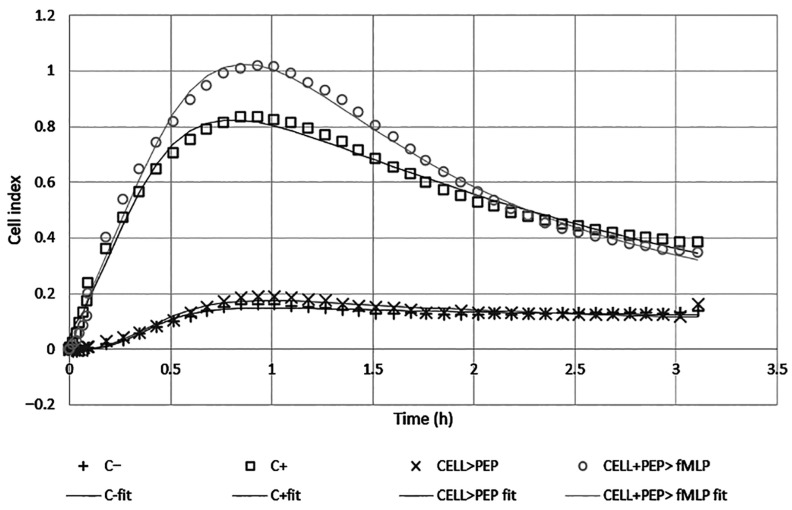
Real-time migration patterns of human neutrophils. The data points in the graph represent the mean value at each time point across replicates for each experimental condition. The lines correspond to the fitted curves derived from the neutrophil migration model equation based on the averaged data.

The ability of ocellatin-LB3 to induce neutrophil chemotaxis was also evaluated. A migration pattern consistent with chemotaxis was observed under conditions in which cells migrated toward N-formylmethionine-leucyl-phenylalanine (fMLP, C+ and CELL + PEP > fMLP), as expected, since fMLP is a well-established chemoattractant used here as a positive control. However, no chemotactic behavior was observed when neutrophils migrated toward the peptide alone (CELL > PEP), as the resulting curve closely resembled that of the negative control (Figure 6).

The migration pattern observed when cells were pre-incubated with the peptide and then exposed to fMLP suggests a potentially additive or synergistic profile, which remains to be confirmed. Notably, between 30 and 90 min, the cell index indicated a higher number of migrated and retained cells than that observed in the profile induced by fMLP alone. However, around the second hour of the experiment, the curves converged to a similar pattern (Figure 6). These findings indicate that ocellatin-LB3 does not act as a direct chemoattractant, but may modulate neutrophil responsiveness to chemotactic stimuli, thereby enhancing fMLP-induced migration. Although biological variability limited the statistical power, the observed trend suggests a potential modulatory effect rather than intrinsic chemotactic activity. Statistically significant differences in cell index were observed only between the negative control and fMLP, and between the peptide and fMLP, confirming the absence of a chemotactic effect induced by the peptide.

Curve fitting and the corresponding statistical analyses revealed significant differences (*p* < 0.05) only between the parameters of the negative control curve and those of the fMLP response curve (positive control).

**Figure 7:**
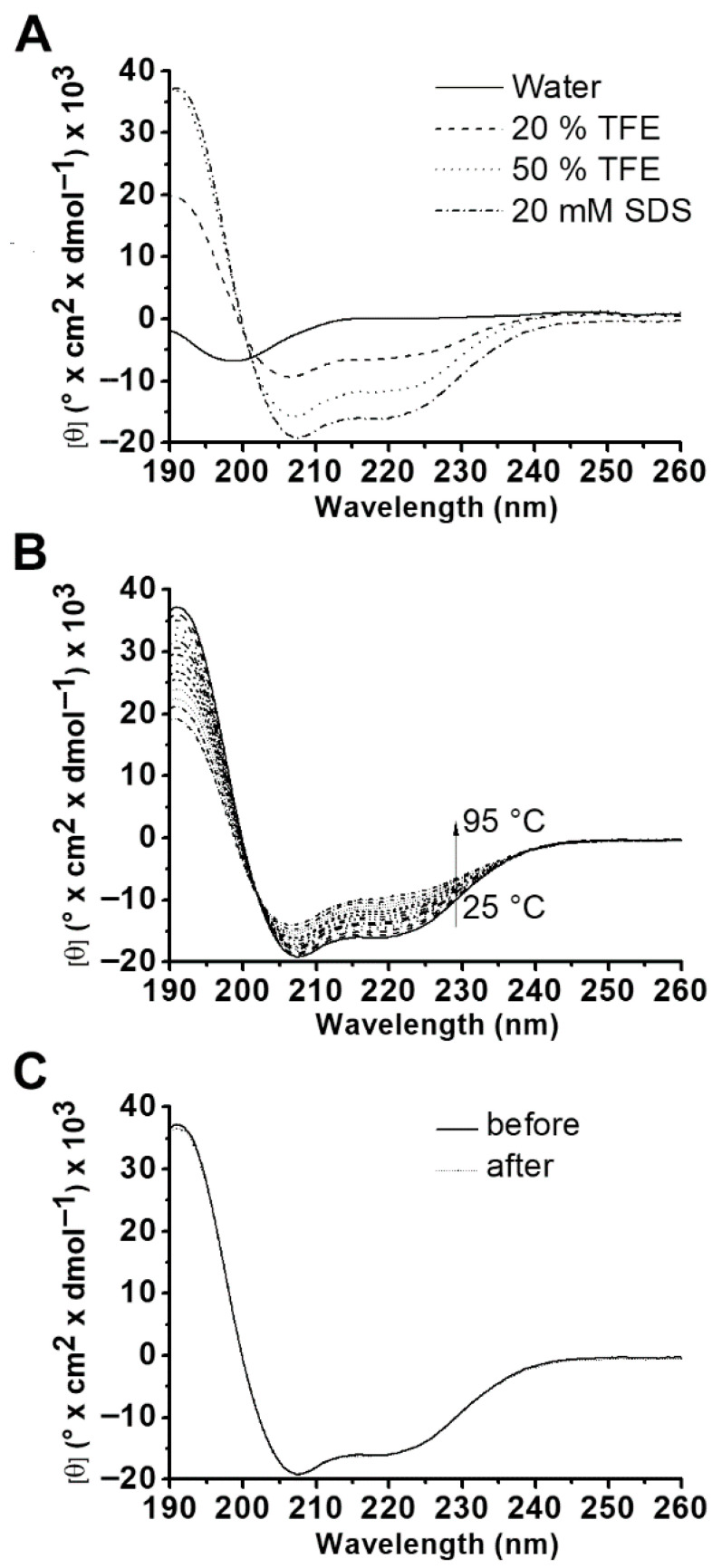
(**A**) Far-UV CD spectra at 25 °C of 32 µM synthetic ocellatin-LB3 in water, 20% (*v*/*v*) TFE, and 50% (*v*/*v*) TFE, and of 42.7 µM synthetic ocellatin-LB3 in 20 mM SDS. (**B**) Thermal denaturation spectra of 42.7 µM synthetic ocellatin-LB3 in the presence of 20 mM SDS, recorded over the temperature range of 25–95 °C at 5 °C intervals. The α-helical content was 63% at 25 °C and 48% at 95 °C. (**C**) Comparison of spectra obtained before heating to 95 °C and after cooling back to 25 °C.

CD spectroscopic analysis of the synthetic peptide (Figure 7A) revealed that, in water, the peptide displays a spectrum indicative of a disordered structure, with a minimum dichroic signal near 199 nm. However, in the presence of 20% TFE, a secondary-structure-inducing solvent, the curve begins to adopt a pattern showing minima near 208 and 222 nm, which become more evident in 50% TFE and are indicative of π-π* and n-π* transitions associated with an α-helical structure [26]. The same pattern was observed when the dichroic spectrum of the peptide was analyzed in 20 mM SDS. The estimated α-helical content was 35% in 20% TFE, 53% in 50% TFE, and 63% in 20 mM SDS.

Thermal denaturation of ocellatin-LB3 in the presence of SDS micelles was also monitored by CD. As the temperature increased from 25 to 95 °C (Figure 7B), the negative bands near 208 and 222 nm decreased in magnitude, indicating a loss of helical content (63% at 25 °C and 48% at 95 °C). The CD spectrum of ocellatin-LB3 after cooling from 95 to 25 °C was similar to that obtained at the beginning of the thermal denaturation experiment at 25 °C (Figure 7C). These data show that the thermal denaturation process was reversible and that the peptide retained the characteristic α-helical spectral profile after returning from 95 to 25 °C.

Complete sequence-specific ^1^H resonance assignments were obtained by combined analysis of 2D TOCSY, COSY, and NOESY spectra. The NMR resonance assignments are presented in Table 2.

**Figure 8:**
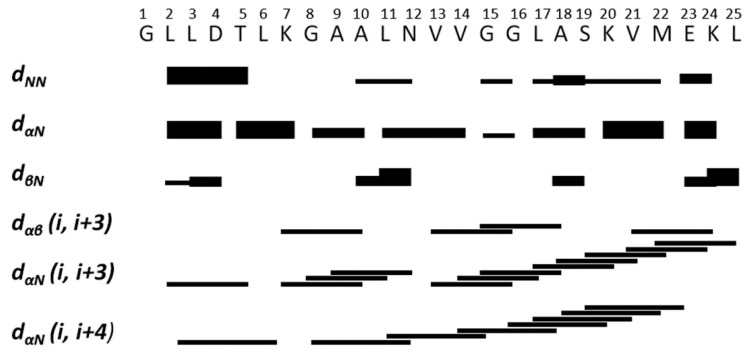
Summary of the sequential and medium-range NOEs observed for synthetic ocellatin-LB3 bound to SDS micelles. Line thickness is proportional to peak intensity and they are classified as strong, medium, or weak, corresponding to upper-bound constraints of 2.8, 3.4, and 5.0 Å, respectively.

Figure 8 summarizes the NOE patterns observed for the peptide and provides a qualitative indication of its secondary structure. The consecutive Hα(i)-NH(i+3) and Hα(i)-NH(i+4) NOEs highlight the presence of a regular α-helix spanning the region from Leu^2^ to Glu^23^.

The three-dimensional structure of ocellatin-LB3 was determined based on the distance restraints derived from the NMR data. A total of 196 distance constraints, of which 53 were medium-range, were used for structure calculation, resulting in an average of 7.84 constraints per residue. The structural statistics are summarized in Table 3.

PROCHECK-NMR [27] was used to evaluate the quality of the energy-minimized structures. As shown in Table 3, the Ramachandran plots confirm the good quality of the NMR models obtained for ocellatin-LB3. This is evidenced by the fact that most ϕ and ψ dihedral angles are located in the most favored region for α-helical conformation (91%).

**Figure 9:**
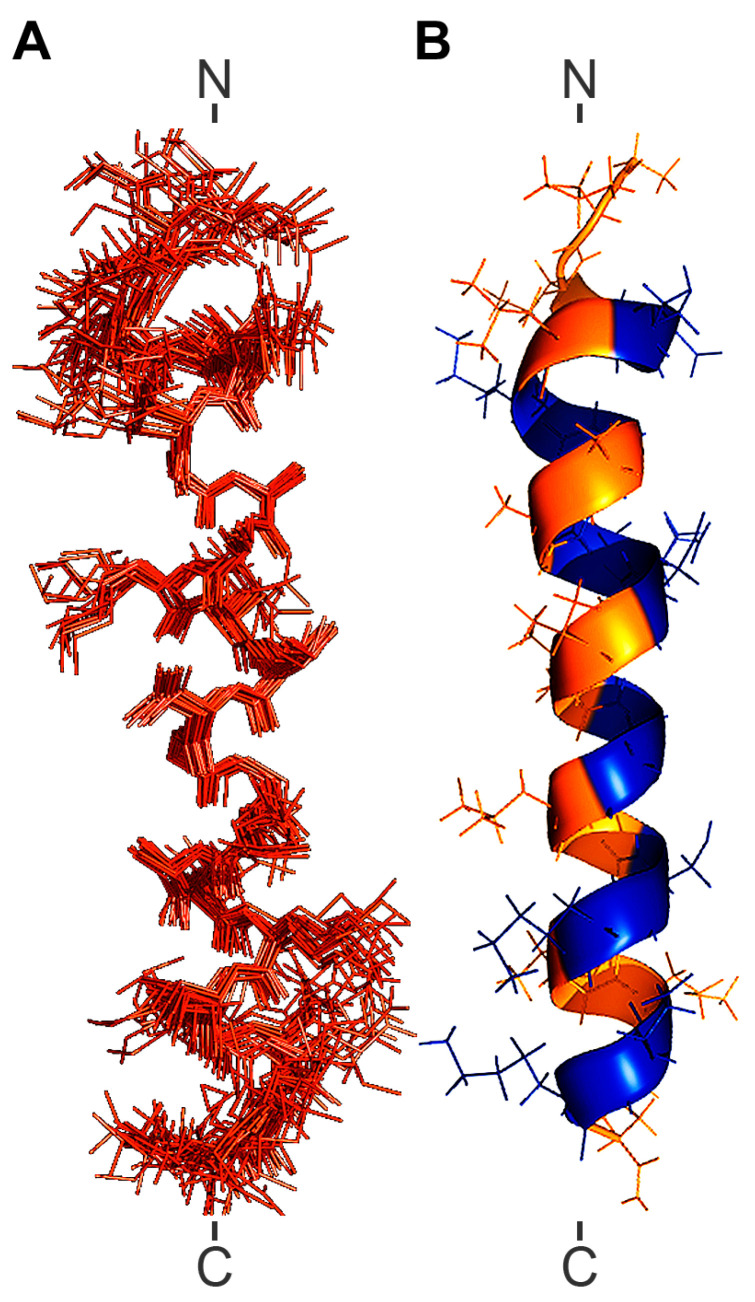
(**A**) Backbone superposition of the final 20 lowest-energy structures of synthetic ocellatin-LB3 bound to SDS micelles over residues 3–24. (**B**) Lowest-energy model of synthetic ocellatin-LB3 bound to SDS micelles. Polar regions are shown in blue and nonpolar regions in orange.

Figure 9 shows the backbone superposition of the final 20 lowest-energy conformers (A), as well as the lowest-energy model (B), of ocellatin-LB3 in the presence of SDS micelles. The structure of ocellatin-LB3 revealed that the peptide adopts a well-ordered helical fold in SDS micelles. The peptide forms an α-helix encompassing residues Leu^3^–Lys^24^ and spanning most of its sequence. The backbone RMSD of the 20 structures was 0.87 ± 0.25 Å (Table 3). This low RMSD value for all backbone atoms confirms the good geometry of the NMR structures. When the RMSD is calculated only for residues Leu^3^-Lys^24^, the value decreases to 0.62 ± 0.24 Å.

**Figure 10:**
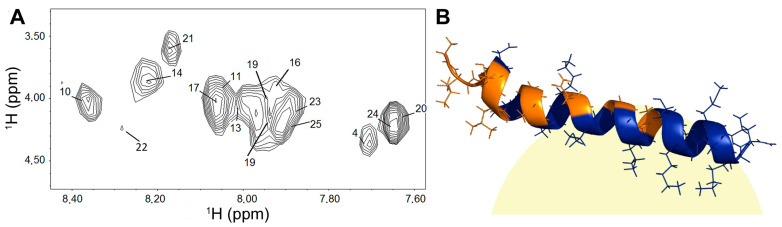
NMR H/D exchange. (**A**) Expansion of the TOCSY spectrum of synthetic ocellatin-LB3 showing the ^1^H chemical shift region of Hα-NH after 10 h 24 min of H_2_O:D_2_O addition. Labels indicate residue numbers. (**B**) Schematic representation of synthetic ocellatin-LB3 bound to an SDS micelle (light yellow sphere). In the peptide, orange indicates exchanged NH residues and blue indicates non-exchanged NH residues.

^1^H NMR experiments were performed to characterize the association of ocellatin-LB3 with SDS-*d*_25_ micelles. A freshly prepared sample of ocellatin-LB3 in H_2_O:D_2_O showed several amide protons protected from solvent exchange. The Hα–NH cross-peaks of residues Leu^2^, Leu^3^, Thr^5^, Leu^6^, Lys^7^, Gly^8^, Ala^9^, Asn^12^, Gly^15^, and Ala^18^ were no longer observed in the first TOCSY spectrum recorded 2 h 24 min after solvent addition, indicating rapid solvent exchange and suggesting that these residues are solvent-exposed. In contrast, the signals corresponding to residues Asp^4^, Ala^10^, Lys^11^, Val^13^, Val^14^, Gly^16^, Leu^17^, Ser^19^, Lys^20^, Val^21^, Met^22^, Glu^23^, Lys^24^, and Leu^25^ were still observable in the spectrum acquired 10 h 24 min after addition of H_2_O:D_2_O (Figure 10A), indicating slow solvent exchange due to protection provided by interaction with the SDS micelle. These results suggest that the peptide lies on the micelle surface, with the C-terminus inserted into the micelle interior (Figure 10B).

## 3. Discussion

We isolated and characterized an antimicrobial peptide that has so far been found exclusively in the skin secretion of *Leptodactylus labyrinthicus*. Ocellatin-LB3 showed selective activity against the Gram-negative bacteria *E. coli*, *K. pneumoniae*, and *P. aeruginosa* at low-to-moderate concentrations, while being essentially inactive against Gram-positive bacteria, since only *Staphylococcus epidermidis* showed slight susceptibility to this peptide (Table 1).

Although there is no consensus on a single mechanism of antimicrobial activity of AMPs, it is generally accepted that their efficacy largely depends on interactions with the target cell membrane [28]. In this context, the proposed membrane-mediated mechanism is supported by indirect evidence, including antimicrobial activity, structural characterization in SDS micelles, and physicochemical properties, but lacks validation by direct membrane permeabilization or depolarization assays; thus, it should be interpreted as indicative rather than definitive. Cationic AMPs are often associated with disruption of membrane stability through interactions with negatively charged phospholipids or LPS in Gram-negative bacteria. In Gram-positive bacteria, interactions may occur with teichoic and teichuronic acids, as well as carboxyl groups of amino acids in the murein layer [29,30,31,32]. Although peptide–bacteria interactions are complex and highly context-dependent, the observed selectivity of ocellatin-LB3 toward Gram-negative bacteria (*E. coli*, *K. pneumoniae*, and *P. aeruginosa*) may be related to differences in membrane composition and surface charge. However, no direct evidence of specific interactions with outer membrane components was obtained. Alternative explanations, including strain-dependent variations in membrane properties, fluidity, or resistance mechanisms, should also be considered.

The physicochemical and biological properties of ocellatin-P1 and the ocellatin-LB3 isoform are comparable (Table 4).

Although substitution of Ser by Gly in ocellatin-LB3 results in subtle changes in hydrophobicity and amphiphilicity, no clear impact on biological activity can be established based on the available data. However, some MIC values obtained in this study for ocellatin-LB3 differ from those reported by [13] for ocellatin-P1. For instance, the *K. pneumoniae* strain used in our study (ATCC 13883) exhibited greater susceptibility to ocellatin-LB3 than the strain used by [13] (KK3 9904) for ocellatin-P1 (MIC = 16 µM vs. 100 µM, respectively). It is important to note that resistance of *K. pneumoniae* to AMPs has been associated with capsule polysaccharides [33], efflux systems [34], and outer membrane proteins involved in adaptive resistance [35], which may vary significantly between strains. Similarly, *P. aeruginosa* showed susceptibility to both ocellatin-P1 (MIC = 100 µM) and ocellatin-LB3 (MIC = 64 µM). For comparison, magainin 2 displayed MIC values of 32 µM against both strains under similar conditions. Ocellatin-LB3 also exhibited activity against *E. coli* (MIC = 4 µM), whereas ocellatin-P1 has been reported to present higher MIC values (25 µM). However, comparisons between ocellatin-LB3 and ocellatin-P1 should be interpreted with caution, as different bacterial strains were used across studies. Strain-specific resistance mechanisms may significantly influence MIC values, making it difficult to attribute differences solely to peptide sequence variations.

Antimicrobial peptides, especially cationic amphibian-derived host-defense peptides, have shown considerable promise as anticancer agents. Their ability to selectively bind to negatively charged cancer cell membranes allows targeted cytotoxicity through membrane disruption or apoptosis induction [36,37]. Amphibian-derived antimicrobial peptides have shown promising anticancer activity. Caerin 1.1 and 1.9, peptides derived from an Australian tree frog, inhibit glioblastoma cell growth and migration, disrupt cell-cycle progression, and alter mitochondrial morphology. They also modulate the expression of proteins associated with metabolism and inflammation [38]. Temporin-SHf, a short cationic peptide, exhibits strong anticancer activity against A549 lung cancer cells by inhibiting proliferation, migration, and angiogenesis, disrupting lysosomal integrity and membrane structure, and inducing apoptosis through a caspase-dependent mitochondrial pathway [39]. Ocellatin-3N, a peptide from *Leptodactylus nesiotus*, inhibits the growth of A549, MDA-MB-231, and HT-29 tumor cells. Despite its limited tumor selectivity, ocellatin-3N represents a promising lead for anticancer peptide development [40].

In the present work, the antiproliferative properties of ocellatin-LB3 were evaluated against HeLa and MCF-7 cancer cell lines, but the peptide was inactive at the tested concentrations except at the highest concentration used (128 µM), which partially inhibited tumor cell growth (Figure 4). The low anticancer activity of ocellatin-LB3 may be improved through the rational design of analogs using this peptide as a structural template. In addition, we demonstrated the trypanocidal potential of ocellatin-LB3 in L6 cells, an immortalized rat muscle cell line, infected with *T. cruzi*. Several AMPs have been investigated for their effects on *T. cruzi*. Apidaecin, magainin II, melittin, and cecropin A have emerged as promising candidates for the treatment of Chagas disease because of their ability to kill *T. cruzi* at low concentrations [41,42]. The oxaborole compound DNDI-6148 has been identified as a potential therapeutic candidate [43]. Nevertheless, progress in the development of effective therapies for Chagas disease remains limited. Overall, various AMPs have exhibited antitrypanosomal properties, positioning them as promising candidates for future clinical evaluation.

AMPs play a dual role in host defense by combining microbicidal activity with immunomodulatory effects. They promote chemotaxis, modulate cytokine production, and help control inflammation through interactions with immune cells, thereby contributing to homeostasis. These properties make AMPs promising therapeutic candidates for infections, inflammatory disorders, and cancer, extending their role beyond direct pathogen elimination [44]. The immunomodulatory effects of ocellatin-LB3 on human neutrophils were evaluated, and although ocellatin-LB3 did not exhibit direct chemotactic activity when administered alone, it enhanced neutrophil migration in response to fMLP. This suggests that the peptide does not function as a classical chemoattractant, but rather modulates neutrophil sensitivity or activation in the presence of a chemotactic signal (Figure 6). This property may be related to a pathway of neutrophil activation via FPR2, leading to increased intracellular Ca^2+^, ERK1/2 phosphorylation, and reactive oxygen species production, but without β-arrestin recruitment or the induction of chemotaxis. Such modulation of chemotactic responsiveness has been described for other host-defense peptides, such as PSMα peptides from *Staphylococcus aureus*, and may represent an immunoregulatory function distinct from direct cell recruitment. This mechanism could open new therapeutic perspectives for the treatment of *S. aureus* infections [45].

α-Helices are present in several structurally characterized amphibian AMPs [46], and many of these peptides are random coils in aqueous solution, adopting α-helical structures upon interaction with the external surface or cytoplasmic membrane of Gram-positive and Gram-negative bacteria [7,29]. CD and NMR analyses showed that ocellatin-LB3 has a random conformation in aqueous solution but is able to adopt an α-helical structure in hydrophobic environments. Indeed, our experimental values for the α-helical content of ocellatin-LB3 were similar to those reported by [20] for ocellatin-LB1 and ocellatin-F1 at the same TFE concentrations. Likewise, the helical content shown by ocellatin-LB3 in 20 mM SDS was similar to that shown by ocellatin-LB1 at the same, and even lower, SDS concentrations. Furthermore, a previous study reported helical contents ranging from 61 to 96% for ocellatin-F1 and several analogs in the presence of 50% TFE [47]. Taken together, these findings indicate that the ability to adopt α-helical structures is a common feature among ocellatins.

In agreement with the CD data, the NMR-derived structure of ocellatin-LB3 reveals an α-helix spanning the region from Leu^3^ to Lys^24^ (Figure 9). The conformational stability of ocellatin-LB3 in the presence of SDS micelles is demonstrated by the thermal denaturation measurements. Figure 7B shows that the peptide loses only part of its helical conformation at 95 °C. Furthermore, it recovers its initial conformation after exposure to high temperature (Figure 7C). This also suggests a strong interaction between the peptide and SDS micelles.

The helical surface of ocellatin-LB3 (Figure 9B) contains four positively charged residues (Lys^7^, Lys^11^, Lys^20^, and Lys^24^) and two negatively charged residues (Asp^4^ and Glu^23^), which may be important for selective interaction with biological membranes. Alignment with previously described ocellatins (Figure 2A) shows that peptide length is a relatively conserved feature, with most of them being 25 residues long. There is a highly conserved N-terminal region in which Lys, Asp, Leu, and Gly are the most conserved amino acids. Asp^4^ is present in all 37 peptides analyzed, and Lys^11^ is present in 36 of them, being conservatively replaced by Arg only in ocellatin-PT5 [48]. Although acidic residues globally reduce the net positive charge, they may also provide structural stability through the formation of salt bridges with basic amino acids [49]. Lysine, leucine, and alanine residues, which are frequent among ocellatins, are known to stabilize or promote α-helix formation [50,51]. Finally, the presence of a glycine residue, located at position 1 in most ocellatins, may confer resistance to aminopeptidases [33].

A C-terminally amidated terminus, another common feature of many antimicrobial peptides, is also present in most ocellatin family peptides described so far, with the exception of ocellatins PT6-PT9 [19,20]. This post-translational modification is apparently important for the adoption and stabilization of an α-helical structure, the increase in net positive charge, and the reduction in susceptibility to carboxypeptidases [52,53].

Figure 10A summarizes the H/D exchange results for ocellatin-LB3 associated with SDS micelles after 10 h 24 min following the addition of H_2_O:D_2_O. Interestingly, one region of the helical structure, composed of residues Leu^2^, Leu^3^, Thr^5^, Leu^6^, Lys^7^, Gly^8^, Ala^9^, Asn^12^, Gly^15^, and Ala^18^, showed a fast NH exchange rate. These results indicate that one portion of the helical structure is exposed to water, whereas the other portion exhibits a slow exchange rate and is therefore protected from solvent. In fact, the signals corresponding to the last seven residues (Ser^19^-Lys^25^), which form the last two turns of the ocellatin-LB3 helix, were not attenuated.

Taken together, these observations support the idea that ocellatin-LB3 interacts with SDS micelles in a preferential orientation. As suggested by the results, the peptide lies on the surface of the micelle, with the C-terminal region inserted into the micelle interior (Figure 10B). The protected region is probably located at the hydrophobic–hydrophilic interface. Charged residues (Asp^4^, Lys^7^, Lys^11^, Lys^20^, Glu^23^, and Lys^24^) exhibited a slow exchange rate, suggesting that they are located in the hydrophilic region of the micelle. On the other hand, Ala^10^, Val^13^, Val^14^, Gly^16^, Leu^17^, Ser^19^, Val^21^, Met^22^, and Leu^25^ are also protected and are probably located in the hydrophobic core of the SDS micelles. The moderate amphipathic character of the peptide, evidenced by the hydrophobic moment value (Table 4), the helical wheel representation (Figure 2B), and the NMR-derived structure (Figure 9), indicates partial segregation of hydrophobic and hydrophilic residues along the helix axis. Although the hydrophobic moment (µH = 0.120) reflects moderate amphipathicity, it supports a structural arrangement in which the C-terminal region preferentially inserts into the micellar environment while maintaining polar residues exposed to the aqueous phase (Figure 10B). Therefore, this structural organization may be associated with the peptide’s biological activity at bacterial membranes. However, this interpretation should be viewed with caution, because SDS micelles, although widely used as membrane-mimetic systems, represent highly simplified and strongly anionic environments that do not fully reproduce the complexity of biological membranes, particularly Gram-negative outer membranes. Accordingly, structural features observed in SDS may not directly reflect peptide behavior in native lipid bilayers.

In our flow cytometric assays, ocellatin-LB3 did not cause detectable alterations in blood cells even after 3 h of treatment. In addition, the peptide did not induce hemolysis even at 128 µM, as confirmed by the hemolytic assay. Because insertion of AMPs into the erythrocyte lipid bilayer depends on their ability to overcome the glycocalyx carbohydrate layer [54], the low affinity of cationic AMPs for zwitterionic membranes may hinder peptide transfer from the glycocalyx to the phospholipid bilayer [55]. As indicated by the Schiffer–Edmundson analysis [56], the α-helical structure of ocellatin-LB3 has a well-defined hydrophobic face (spanning approximately half of the helix cross-section, although this face is interrupted by Lys^20^ and Lys^24^ (Figure 2B). Nevertheless, the hydrophilic face is less clearly defined than in other AMPs, with acidic and basic residues distributed throughout the sequence. Amphiphilicity has been suggested to be important both for hemolytic activity and for antibacterial efficacy against Gram-positive bacteria [57,58]. Accordingly, the relatively low ‹µH› value of this peptide may help explain the limited biological activities observed in those cells.

Considering that the amino acid sequence of an AMP is a major determinant of its three-dimensional structure, it seems reasonable that the maintenance of residues conferring key structural features has been favored over evolutionary time, since evolutionary pressures on proteins tend to preserve secondary-structure-related properties such as hydrophobicity, backbone conformational preferences, and side-chain bulk [59]. In addition, the high degree of primary-structure conservation observed among ocellatins may indicate the presence of α-helical motifs in many of them, as previously suggested for some members of this family [20,47].

## 4. Material and Methods

### 4.1. Chemicals and Microorganisms

Only analytical-grade reagents from commercial suppliers were used throughout this work, and all solutions were prepared with ultrapure (Type 1) water (Milli-Q system, Merck Millipore, Burlington, MA, USA). Reagents and solvents for protein sequencing were purchased from Applied Biosystems (Waltham, MA, USA) and from Shimadzu Scientific Instruments (Kyoto, Japan). Solvents for chromatographic procedures were HPLC grade and obtained from several commercial sources. All natural Fmoc amino acids and Rink-amide-MBHA resin were purchased from Synbiosci (Frederick, MD, USA) and Novabiochem (Merck Millipore, Burlington, MA, USA). Solvents and reagents for peptide synthesis were obtained from Sigma-Aldrich Co. (St. Louis, MO, USA), Fluka (Buchs, Switzerland), and Hexis Científica (Jundiaí, SP, Brazil). The bacterial strains *Escherichia coli* (ATCC 25922), *Klebsiella pneumoniae* (ATCC 13883), *Pseudomonas aeruginosa* (ATCC 27853), *Staphylococcus aureus* (ATCC 25923), *Staphylococcus epidermidis* (ATCC 12228), and *Enterococcus faecalis* (ATCC 29212) were obtained from LACEN (Central Public Health Laboratory, Brasília, DF, Brazil). Blood for the hemolysis and flow cytometry assays was obtained from a healthy donor.

### 4.2. Specimen Collection and Skin Secretion Harvesting

Adult specimens of *Leptodactylus labyrinthicus* (*n* = 2) were captured by visual encounter and acoustic recognition in Luziânia, Goiás, Brazil, and transported in damp boxes to the Laboratory of Toxinology at the University of Brasília, where they were maintained in captivity. Skin secretions were obtained by electrical stimulation using a device with two electrodes, applying an alternating current of approximately 110 V at 60 Hz to the dorsal surface, followed by rinsing with ultrapure water. The material was collected in glass containers, freeze-dried, and stored at −20 °C until use. All procedures were approved by the Ethics Committee on Animal Use of the University of Brasília (CEUA, number 110, 16 December 2019).

### 4.3. Peptide Purification

Aliquots of 5 mg of freeze-dried skin secretion were dissolved in 220 µL of 0.1% (*v*/*v*) TFA/water, centrifuged for 5 min at room temperature, and the supernatant was subjected to RP–HPLC on a C_8_ column (Shim-pack CLC–C8(M) 150 x 4.6 mm, Shimadzu Corporation, Kyoto, Japan) equilibrated with 0.1% (*v*/*v*) TFA/water (solvent A). After a 5 min initial wash with solvent A, elution was performed with linear gradients of 0.1% TFA/acetonitrile (solvent B) at a flow rate of 1 mL/min as follows: 0–55% in 55 min, 55–100% in 5 min, followed by a final 5 min wash with 100% solvent B. Absorbance was monitored at 216 nm, and the fractions were manually collected, dried in a centrifugal evaporator, and pooled for antibacterial testing as described below. After identification of the active fractions, one fraction of them, containing the ocellatin-P1 isoform was pooled from seven chromatographic runs and diluted in 0.1% (*v*/*v*) TFA/water. It was then injected onto a C_18_ column (Shim-pack VP-ODS 150 x 4.6 mm, Shimadzu Corporation, Kyoto, Japan) equilibrated with solvent A. After a 5 min initial wash with solvent A, elution was performed at a flow rate of 1 mL/min as follows: 0–30% in 10 min, 30–50% in 20 min, 50–100% in 5 min, followed by a final 5 min wash with 100% solvent B. Fractions were collected while monitoring absorbance at 216 nm and were then dried under vacuum for subsequent analysis.

### 4.4. Structural Analysis

Mass analysis of the native ocellatin-P1 isoform was performed by MALDI-TOF MS using an Autoflex II TOF/TOF mass spectrometer (Bruker Daltonics, Bremen, Germany) in the 0–4000 m/z range, using positive reflector mode and CHCA as matrix. Calibration was performed with a mixture of angiotensin II (Mr = 1046.54), angiotensin I (Mr = 1296.68), substance P (Mr = 1347.74), bombesin (Mr = 1619.82), adrenocorticotropic hormone fragment 1–17 (Mr = 2093.09), and adrenocorticotropic hormone fragment 18–39 (Mr = 2465.20). Amino acid sequencing of the purified peptide was achieved by Edman degradation using a 477A automatic sequencer (Applied Biosystems, Foster City, CA, USA) modified as described in [60]. The presence of a C-terminal amidation in the molecule was evaluated by Fischer-Speier esterification, following the method described in [61].

Clustal Omega [62] was used for multiple sequence alignment (https://www.ebi.ac.uk/jdispatcher/msa/clustalo (accessed on 10 June 2025)). The theoretical monoisotopic molecular mass and isoelectric point were calculated from the sequence using the Compute pI/Mw tool [63] (https://web.expasy.org/compute_pi/ (accessed on 10 June 2025)). Mean hydrophobicity (‹H›) and mean hydrophobic moment (‹µ_H_›) were calculated according to the Eisenberg consensus hydrophobicity scale [64]. Finally, NetWheels (https://neutrophil-proteome.shinyapps.io/netwheels/ (accessed on 12 June 2025)) [65] was used to obtain a helical wheel projection of the peptide.

### 4.5. Peptide Synthesis

Ocellatin-LB3 was manually synthesized according to the standard Nα-Fmoc protecting-group strategy [66], following the methodology described in [67]. The side-chain protecting groups Boc and Trt were used for Lys and Asn, respectively. After the coupling of the C-terminal amino acid to the resin (Rink-amide-MBHA), the successive α-amino group deprotection and neutralization steps were performed in 20% piperidine/DMF for 20 min. The amino acids were coupled at threefold excess using DIC/HOBt in 50% (*v*/*v*) DCM/DMF and, when necessary, TBTU/HOBt/DIEA in 50% (*v*/*v*) DCM/NMP. After a 2 h coupling time, the ninhydrin (2,2-dihydroxyindane-1,3-dione) test was performed to assess reaction completeness. Cleavage from the resin and removal of the side-chain protecting groups were carried out simultaneously using 90% TFA, 5% p-cresol, and 5% water for 2 h. In this procedure, the crude peptide was precipitated with anhydrous ethyl ether, separated from soluble non-peptide material by centrifugation, extracted into 0.045% (*v*/*v*) TFA/H_2_O (solvent A), and lyophilized.

Purification of the synthesized peptide was accomplished by HPLC on a reversed-phase C_18_ column (Beckman C_18_ 1 x 25 cm) using a linear gradient 10–40% of 0.036% (*v*/*v*) TFA/acetonitrile (solvent B) over 90 min at a flow rate of 5 mL/min. UV detection was performed at 220 nm. The peptide sequence was confirmed by Edman degradation using a PPSQ-31A/33A protein sequencer (Shimadzu Scientific Instruments, Kyoto, Japan).

### 4.6. Antimicrobial Assays

Antibacterial assays were performed according to the CLSI M7-A6 [68] with some modifications. Briefly, the bacterial cultures were grown in 7 mL of Mueller–Hinton (MH) medium (HiMedia, Mumbai, Maharashtra, India) under continuous shaking at 37 °C until the optical density (OD) at 625 nm reached a range of 0.08–0.10. The cultures were then diluted 1:10 in MH medium, and the inoculum was prepared by a further 1:20 dilution to obtain 5 × 10^5^ cells/mL. Each fraction was dissolved in Milli-Q water, and 50 μL of the resulting solution was transferred to wells of a flat-bottom microplate. An equal volume (50 μL) of the bacterial suspension was then added. After incubation at 37 °C for 24 h, OD readings at 625 nm were recorded using a Multiskan FC microplate reader (Thermo Fisher Scientific, San Jose, CA, USA). Formaldehyde at 0.8% (*v*/*v*) was used as a control for absence of bacterial growth, whereas Milli-Q water served as the reference for full growth (100%); both were incubated alongside the bacterial suspensions. Magainin 2 was used as a model AMP and tested against *P. aeruginosa* ATCC 27853 and *K. pneumoniae* ATCC 13883.

### 4.7. Toxicity in Blood Cells

To evaluate toxicity toward red and white blood cells, cell counts were performed using a Cell-Dyn Ruby hematology analyzer (Abbott Laboratories, Abbott Park, IL, USA). O^+^ blood from a healthy male donor had the plasma removed by centrifugation (5 min at 3000 rpm) and was washed three times with saline (0.9% NaCl), after which the cells were resuspended in the same solution. Aliquots of 750 µL of the cell suspension were incubated in triplicate under three conditions: (1) without addition of any substance (negative control), (2) with 1% (*v*/*v*) Triton X-100 (positive control), and (3) with synthetic ocellatin-LB3 at a final concentration of 64 µM. Cell counts obtained after 0, 30, and 90 min were used to compare treatments by unpaired *t*-tests using Prism 5.03 software (GraphPad Software, San Diego, CA, USA). The procedure was approved by the Human Ethics Committee of the University of Brasília (CEP-FM, number 45, 21 July 2010).

### 4.8. Hemolytic Assays

The hemolytic assay was based on [69]. Briefly, fresh human blood (O^+^) from a healthy male donor was collected in EDTA and washed with saline buffer (0.01 M Tris-HCl, pH 7.4, containing 0.15 M NaCl and 0.01 M CaCl_2_). Erythrocytes were separated from plasma by sedimentation and resuspended in the same buffer to obtain a 1% (*v*/*v*) suspension. To determine the HC_50_ (the concentration causing 50% hemolysis), aliquots of the cell suspension were incubated in triplicate with serial dilutions of synthetic ocellatin-LB3 ranging from 128 to 0.25 µM. Triplicate samples of cell suspension alone (0% lysis, negative control) and with 1% (*v*/*v*) Triton X-100 (100% lysis, positive control) were also included. After 1 h at 37 °C, the samples were centrifuged at 3000× *g* for 2 min. Aliquots of the supernatant were transferred to 96-well microplates, and absorbance was measured at 405 nm using a Multiskan FC microplate reader (Thermo Fisher Scientific, San Jose, CA, USA). The percentage of hemolysis at each peptide concentration was calculated according to the formula described in [70]:

$$\frac{A_{405\;nm,\;peptide}-A_{405\;nm,\;buffer}}{A_{405\;nm,\;detergent}-A_{405\;nm,\;buffer}}\times 100$$

The study protocol received approval from the Human Ethics Committee of the University of Brasília (CEP-FM, number 45, 21 July 2010).

### 4.9. Antiproliferative Assays

HeLa (human cervical adenocarcinoma, ATCC CCL-2) and MCF-7 (human breast adenocarcinoma, ATCC HTB-22) cells were cultured at 37 °C with 5% CO_2_ in Dulbecco’s Modified Eagle Medium (DMEM) (Sigma-Aldrich, St. Louis, MO, USA), supplemented with 100 IU/mL penicillin, 100 μg/mL streptomycin, and 10% (*v*/*v*) fetal bovine serum (Invitrogen, Waltham, MA, USA). For the assays, cells (10 × 10^3^/well) were seeded into 96-well microplates (TPP Techno Plastic Products AG, Trasadingen, Switzerland) and incubated overnight in complete DMEM. After this period, 200 µL of peptide solution (1–128 µM) prepared in complete medium was added to each well. After 24 h, cell viability was evaluated by the MTT assay (3-(4,5-dimethylthiazol-2-yl)-2,5-diphenyl tetrazolium bromide; Invitrogen, Waltham, MA, USA). Briefly, 15 µL of MTT solution (5 mg/mL in PBS) and 135 µL of DMEM were added to each well, followed by incubation for 3 h at 37 °C with 5% CO_2_. The medium was then removed, and 100 µL of dimethyl sulfoxide (DMSO) was added, and absorbance was measured at 595 nm using a Multiskan FC microplate reader Thermo Fisher Scientific, San Jose, CA, USA).

### 4.10. Anti-T-Cruzi Activity

L6 cells (rat skeletal muscle) were grown in 96-well plates at a density of 1000 cells/well in RPMI 1640 medium (Gibco, Waltham, MA, USA) supplemented with 10% heat-inactivated fetal bovine serum (FBS), maintained at 37 °C in a 5% CO_2_ atmosphere, and infected with *T. cruzi* trypomastigotes at a 30:1 ratio for 24 h. Non-internalized parasites were removed by washing, and the infected cells were treated with the compound (1.5–0.2 µM) for 72 h. Analyses were performed using panoptic staining. The infection inhibition rate was determined by comparing the percentage of infected cells in the treated groups with that of the untreated control group.

### 4.11. Real-Time Migration Assays (Chemotaxis on Neutrophils)

#### 4.11.1. Cell Preparation Method and Data Collection

Neutrophils were obtained from peripheral blood collected from three healthy donors and isolated using a Percoll density gradient. The cells were subjected to real-time migration assays to assess chemotaxis using plates containing two chambers (an upper and a lower chamber) separated by an electrode oriented toward the lower chamber. This electrode contains pores that allow active cell passage and enable quantification of both cells that migrated and cells that remained adhered to the electrode after migration. Aliquots containing 6600 cells/µL in HBSS buffer were placed in the upper chamber, and the agent to be evaluated for chemotactic potential was placed in the lower chamber according to the conditions described in the experimental design. The RTCA device (Agilent Technologies, Inc., Santa Clara, CA, USA) was activated to monitor neutrophil migration through the micropores in the electrode, recording impedance in real time every 5 min for 3 h.

Experimental conditions were as follows: blank (HBSS in both chambers, no cells)—HBSS > HBSS; negative control (spontaneous migration without chemoattractants—neutrophils in HBSS in the upper chamber, HBSS in the lower chamber)—CELL > HBSS; positive control with bacterial peptide—fMLP (neutrophils in HBSS in the upper chamber, fMLP in the lower chamber)—CELL > fMLP or C+; chemotactic effect of the peptide (neutrophils in HBSS in the upper chamber, peptide in the lower chamber)—CELL > P; and comparative effect between peptide and fMLP (neutrophils in peptide solution in the upper chamber, fMLP in the lower chamber)—CELL + P > fMLP.

#### 4.11.2. Data Analysis

The “cell index” values, which represent the impedance of each well and are proportional to the number of cells in contact with the electrode after migration through the pores of the plate, were recorded every 5 min.

These values were background-subtracted using the blank and then analyzed by descriptive statistics, direct point-to-point statistical comparison at each time point, statistical comparison of the area under the curve (AUC), mathematical modeling and AI-based parameterization of the migration curves according to the following equation:

$$t\left(x\right)=a{\;e}^{\left(\left(b-c\left(x+d\right)\right){\;e}^{\left(f\left({\;x}^{\left(g-1\right)}+d\right)\left(h-\left(x+d\right)\right)\right)}\right)\;}e^{-k\;x^{g}}$$

The equation was derived from a combination of the Gompertz and Boltzmann functions and allowed an appropriate fit of the curves obtained under various experimental conditions.

Data from each replicate within each experimental condition were used to generate cell index versus time curves using SciDavis software (version 2.7). Each curve was fitted to the aforementioned equation, and the parameters a, b, c, d, f, g, h, and k were recorded for curves showing a good correlation (r^2^ > 0.99).

Statistical comparison of curve parameters: each fitted parameter was compared across the different experimental conditions.

The protocol described above was approved by the Human Ethics Committee of the University of Brasília (CEP-FM, number 45, 21 July 2010).

### 4.12. Circular Dichroism

Circular dichroism spectra were obtained using a J-815 spectropolarimeter (JASCO Corporation, Tokyo, Japan) equipped with a Peltier-type temperature-controlled cuvette holder. Samples of synthetic ocellatin-LB3 were prepared at a final concentration of 32 μM in ultrapure water, 20% (*v*/*v*) TFE, and 50% (*v*/*v*) TFE, and at a final concentration of 42.7 μM in 20 mM SDS. A 0.1 cm path-length quartz cell, 0.1 nm step resolution, scan speed of 50 nm/min, response time of 8 s, and bandwidth of 1 nm were generally used. Spectra were acquired at 25 °C in the far-UV region (190–260 nm) as the average of 3–4 scans. For the thermal denaturation curve in SDS, measurements were performed at 5 °C intervals over the temperature range of 25–95 °C.

Following subtraction of solvent effects, the observed ellipticities, *θ* (milidegree), were converted to molar ellipticity ([*θ*] (cm^2^ dmol^−1^)) using the following equation [71]:

$${\left[\theta \right]}_{\lambda }=\frac{\theta_{\lambda }\times MRW}{10\times l\times c}$$

where *θ* is the ellipticity in millidegrees at wavelength λ, *MRW* is the mean residue mass, obtained by dividing the molecular mass by the number of peptide bonds (n − 1, where n is the number of amino acid residues), *l* is the optical path length in cm, and ***c*** is the concentration in g/mL.

The α-helical secondary structure content (fractional helicity, *f*_H_) was estimated for each solvent using the following equation [72]:

$$f_{H}=\frac{{\left[\theta \right]}_{208}-4000}{\left(-33,000-4000\right)}$$

### 4.13. NMR Spectroscopy

To investigate the conformational properties of ocellatin-LB3, its three-dimensional structure was determined by ^1^H NMR in the presence of SDS-*d*_25_ micelles. SDS micelles have a negatively charged surface and are considered a suitable model for anionic membranes such as bacterial membranes [73,74]. Samples for NMR measurements were prepared by dissolving the synthetic peptide to 1 mM in 100 mM SDS-*d*_25_ solution, pH 4.3, containing 10% D_2_O and 0.5% TMSP-*d*_4_ as the reference standard (0 ppm). Two-dimensional ^1^H-TOCSY (mixing time = 60 ms), NOESY (mixing time = 200 ms), and COSY experiments were acquired at 36 °C on an AVANCE III 500 spectrometer (Bruker Daltonics, Bremen, Germany) operating at 11.75 T. Typically, spectra were recorded with 56 scans for ^1^H-TOCSY and 88 scans for ^1^H-NOESY, using 2048 data points and 256 t1 increments. Complete assignment of the backbone and side-chain ^1^H resonances of ocellatin-LB3 was performed using standard sequential assignment procedures [75]. All NMR data were processed using NMRPipe version 3.5 and NMRViewJ version 5.0.4 [76,77].

To obtain interproton distance constraints, NOESY cross-peak volumes were measured and calibrated with respect to the cross-peak volume of geminal β-protons (1.8 Å). The resulting NOE values were classified as strong, medium, and weak, corresponding to upper-bound distances of 2.8, 3.4, and 5.0 Å, respectively.

### 4.14. Molecular Modeling

Structure calculations were performed with XPLOR-NIH [78,79] version 2.28 using a simulated annealing protocol [80]. The 20 lowest-energy structures (from a total of 200 calculated structures) were selected to represent the three-dimensional solution structure of the peptide. The quality of the energy-minimized structures was analyzed using PROCHECK-NMR [27] version 3.4. Final structures were visualized using PyMOL [81] version 1.2r1.

### 4.15. Orientation of the Peptide in SDS-d_25_ Micelles

To obtain an indication of the orientation of ocellatin-LB3 in SDS micelles, NMR H/D exchange experiments were performed and the exchange of peptide backbone amide protons was monitored. ^1^H TOCSY NMR experiments were recorded on an AVANCE III 500 spectrometer (Bruker Daltonics, Bremen, Germany) as described above. The peptide-incorporated SDS-*d*_25_ sample, containing 1 mM synthetic ocellatin-LB3 and 100 mM SDS-*d*_25_, was lyophilized, and 50% D_2_O:50% H_2_O was added immediately before NMR acquisition. A series of TOCSY spectra was then recorded every 2 h over a 10 h period, starting 24 min after sample preparation.

## 5. Conclusions

In this work, we report the isolation, purification, and biological characterization of a novel peptide from the skin secretion of *Leptodactylus labyrinthicus*. In addition, the three-dimensional structure of an ocellatin family member was determined, revealing an amphipathic α-helical conformation in a membrane-mimetic environment. The combined structural and physicochemical analyses, together with the antimicrobial activity data, support a possible membrane-associated mode of action. However, this interpretation is based on indirect evidence, including structural characterization in SDS micelles, and was not validated by direct membrane permeabilization or depolarization assays. Although ocellatin-LB3 exhibited antimicrobial activity, its selectivity and biological effects remain to be further elucidated, particularly in relation to strain-dependent variability and potential interactions with complex membrane systems. Overall, further studies are needed to validate the potential of ocellatin peptides for the development of antibiotic agents through structural optimization and biological activity improvement, as well as to explore their immunomodulatory potential in wound-healing assays.

## Figures and Tables

**Table 1:** Minimal inhibitory concentrations of synthetic ocellatin-LB3 against reference Gram-negative and Gram-positive bacterial strains.

Bacteria	MIC (µM)
*Escherichia coli* ATCC 25922	4
*Klebsiella pneumoniae* ATCC 13883	16
*Pseudomonas aeruginosa* ATCC 27853	64
*Staphylococcus epidermidis* ATCC 12228	>128
*Staphylococcus aureus* ATCC 25923	n.a.
*Enterococcus faecalis* ATCC 29212	n.a.

n.a. = no activity found.

**Table 2:** ^1^H chemical shifts (ppm) of synthetic ocellatin-LB3 in 100 mM SDS-*d*_25_.

Residue	δ_NH_	δ_Hα_	δ_Hβ_	δ_H others_
Leu^2^	8.65	4.16	1.65, 1.67	*γ* 1.57
Leu^3^	8.35	4.06	1.75, 1.56	*γ* 1.83
Asp^4^	7.73	4.37	2.78, 2.82	-
Try^5^	7.98	4.38	4.01	*γ* 1.24
Leu^6^	8.08	4.15	1.65, 1.55	*γ* 1.85, *δ* 1.9
Lys^7^	8.34	4.11	1.61, 1.86	*γ* 1.47
Gly^8^	8.25	4.05, 3.88	-	-
Ala^9^	7.92	4.39	1.53	-
Ala^10^	8.39	4.02	1.49	-
Lys^11^	8.04	3.93	1.95, 173	*γ* 1.38, *δ* 1.63, *ε* 3.00
Asn^12^	7.79	4.60	2.94, 2.91	-
Val^13^	8.02	4.06	2.26	*γ* 1.14, *γ* 1.01
Val^14^	8.19	3.85	2.25	*γ* 1.06, *γ* 1.02
Gly^15^	8.38	3.87	-	-
Gly^16^	7.91	3.95	-	-
Leu^17^	8.05	4.04	1.73	-
Ala^18^	8.63	3.95	1.53	-
Ser^19^	7.95	4.21	4.00	-
Lys^20^	7.63	4.15	1.83, 1.65	*γ* 1.52
Va^l21^	8.16	3.59	2.23	*γ* 1.06, *γ* 0.95
Met^22^	8.31	4.21	2.15, 1.97	*γ* 2.62, *δ* 2.76
Glu^23^	7.88	4.11	2.22, 2.2	*γ* 2.59, *γ* 2.47
Lys^24^	7.66	4.23	1.98, 1.71	*γ* 1.53, *δ* 1.71, *ε* 2.98
Leu^25^	7.89	4.23	1.78, 1.74	*δ* 1.60

**Table 3:** Statistics for the best 20 NMR structures of synthetic ocellatin-LB3.

**Total Distance Restrains**	**196**
Intraresidue	57
Sequential	46
Medium (1 < |i − j| < 5)	53
Hydrogen Bonds	40
**PROCHECK—Ramachandran plot analysis**	**%**
Most favoured region	91
Additionally allowed region	4.5
Generously allowed region	4.5
Disallowed region	0
**RMSD**	**Å**
Backbone (all)	0.87 ± 0.25
Backbone (residues 3–24)	0.62 ± 0.24

**Table 4:** Physicochemical properties of ocellatin-P1 and ocellatin-LB3.

Peptide	Mr	Q	pI	‹H›	‹µ_H_›
Ocellatin-P1	2542.06	+3	9.53	−0.053	0.128
Ocellatin-LB3	2512.03	+3	9.53	−0.036	0.120

Mr = relative molecular mass; Q = charge (at neutral pH); pI = isoelectric point; ‹H› = mean hydrophobicity; ‹µ_H_› = mean hydrophobic moment. The pI calculation does not consider C-terminal amidation.

## Data Availability

The original contributions presented in this study are included in the article. Further inquiries can be directed to the corresponding author.

## Abbreviations

The following abbreviations are used in this manuscript:

AMPs	Antimicrobial peptides
Boc	N-tert-butoxycarbonyl
CHCA	Alpha-cyano-4-hydroxycinnamic acid
DCM	Dichloromethane
DIC	N,N’-Diisopropylcarbodiimide
DIEA	Diisopropyl ethylamine
DMF	Dimethylformamide
fMLP	N-formylmethionine-leucyl-phenylalanine
Fmoc	9-Fluorenylmethyl-oxycarbonyl
HOBt	N-Hydroxybenzotriazole
MIC	Minimum inhibitory concentration
NMP	N-Methylpyrrolidone
Rink-amide-MBHA	4-(2′,4′-Dimethoxyphenyl-Fmoc-aminomethyl)-phenoxyacetamido-norleucyl-(4-methylbenzhydrylamine)
RMSD	Root mean square deviation
RP-HPLC	Reversed-phase high-performance liquid chromatography
TBTU	2-(1H-Benzotriazole-1-yl)-1,1,3,3-Tetramethylaminium tetrafluoroborate
TFE	2,2,2-Trifluoroethanol
TMSP-*d*_4_	Sodium-2,2,3,3-tetradeutero-3-trimethylsilylpropionate
TOCSY	Total correlation spectroscopy
Trt	Trityl
